# Association between dual sensory impairment and risk of mortality: a cohort study from the UK Biobank

**DOI:** 10.1186/s12877-022-03322-x

**Published:** 2022-08-01

**Authors:** Xinyu Zhang, Yueye Wang, Wei Wang, Wenyi Hu, Xianwen Shang, Huan Liao, Yifan Chen, Katerina V. Kiburg, Yu Huang, Xueli Zhang, Shulin Tang, Honghua Yu, Xiaohong Yang, Mingguang He, Zhuoting Zhu

**Affiliations:** 1 Department of Ophthalmology, Guangdong Academy of Medical Sciences, Guangdong Provincial People’s Hospital, Guangzhou, China; 2 Department of Ophthalmology, Shanghai General Hospital, Shanghai, China; 3 State Key Laboratory of Ophthalmology, Zhongshan Ophthalmic Center, Sun Yat-Sen University, Guangzhou, China; 4 Centre for Eye Research, University of Melbourne, East Melbourne, Victoria, Australia; 5 Neural Regeneration Group, Institute of Reconstructive Neurobiology, University of Bonn, Bonn, Germany; 6 John Radcliffe Hospital, Oxford University Hospitals NHS Foundation Trust, Oxford, UK

**Keywords:** Mortality, Sensory impairment, Visual impairment, Hearing impairment, UK Biobank

## Abstract

**Background:**

Dual sensory impairment is affecting over 10% of older adults worldwide. However, the long-term effect of dual sensory impairment (DSI) on the risk of mortality remains controversial. We aim to investigate the impact of single or/and dual sensory impairment on the risk of mortality in a large population-based sample of the adult in the UK with 14-years of follow-up.

**Methods:**

This population-based prospective cohort study included participants aged 40 and over with complete records of visual and hearing functions from the UK Biobank study. Measurements of visual and hearing functions were performed at baseline examinations between 2006 and 2010, and data on mortality was obtained by 2021. Dual sensory impairment was defined as concurrent visual and hearing impairments. Cox proportional hazards regression models were employed to evaluate the impact of sensory impairment (dual sensory impairment, single visual or hearing impairment) on the hazard of mortality.

**Results:**

Of the 113,563 participants included in this study, the mean age (standard deviation) was 56.8 (8.09) years, and 61,849 (54.5%) were female. At baseline measurements, there were 733 (0.65%) participants with dual sensory impairment, 2,973 (2.62%) participants with single visual impairment, and 13,560 (11.94%) with single hearing impairment. After a follow-up period of 14 years (mean duration of 11 years), 5,992 (5.28%) participants died from all causes. Compared with no sensory impairment, dual sensory impairment was significantly associated with an estimated 44% higher hazard of mortality (hazard ratio: 1.44 [95% confidence interval, 1.11–1.88], *p* = 0.007) after multiple adjustments.

**Conclusions:**

Individuals with dual sensory impairment were found to have an independently 44% higher hazard of mortality than those with neither sensory impairment. Timely intervention of sensory impairment and early prevention of its underlying causes should help to reduce the associated risk of mortality.

**Supplementary Information:**

The online version contains supplementary material available at 10.1186/s12877-022-03322-x.

## Background

Worldwide, at least 2.2 billion people were affected by visual impairment (VI) and 1.5 billion people by hearing impairment (HI) in 2019 as reported by the WHO [1, 2]. As the global population grows, nearly one in every four people is predicted to be suffered from hearing loss, and 237 million people are estimated to have moderate to severe VI, by 2050 [2, 3]. Subject to the worldwide epidemiological transition of aging, dual sensory impairment (DSI), defined as concurrent VI and HI, has been estimated to affect over 10% of older adults aged 80 years and over [4]. DSI results in a high hospitalization rate and significant healthcare cost [5], presenting a fundamental challenge to public health.

VI and HI impacts various aspects of an individual's life, while the adverse outcomes of DSI are even more wide-reaching with stronger associations. It was previously reported that people with DSI are exposed to an increased risk of co-morbidities (cognitive impairment, dementia, depression, etc.) when compared with those with single VI or HI [6–8]. Moreover, DSI is related to a higher risk of severe life-threatening consequences, including falls and accidental injury, and individuals with DSI also face difficulty accessing healthcare services [9–11]. The combined effects of reduced quality of life, increased comorbidities, barriers to accessing healthcare, and higher incidence of accidents may all contribute to an increased risk of mortality among adults with DSI.

Prior investigations have reported a significant association between single sensory impairment, VI or HI, with mortality [12–14]. However, only a few studies, which have been summarized in Additional file 1: Supplemental Table 1 , evaluated the increased risk of the association with DSI and mortality, with inconsistent results [15–22]. For example, Lee, J David et al. reported that self-reported dual sensory impairment, both mild/moderate and severe, was found significantly associated with mortality in 116,796 men and women over 18 years old [20]. However, Yamada, Yukari et al. found that objectively determined minimum to severe impairment in both senses were not significantly associated with mortality in 2,851 participants with a mean age of 83.6 years [21]. Difference across the small number of prior studies may be due to: unstandardized subjective measurements for sensory impairments, small sample sizes and highly selected populations without enough representation. Therefore, to address the abovementioned gap, in the present study, we analyzed data from a large population-based longitudinal setting, the UK Biobank and investigated the association of VI, HI, and DSI with 14-year mortality. We hypothesize that VI and HI are positively associated with 14-year mortality, and DSI even have a higher risk than single sensory impairment.

## Methods and materials

### Study design and population

From March 13, 2006 to December 1, 2010, the UK Biobank recruited 502,462 participants aged 40 to 69 years across the United Kingdom, with the involvement of 22 assessment centres in England, Scotland and Wales [23]. For collection of baseline information, each participant completed a questionnaire, took physical measurements, and provided biological samples. Northwest Multi-Centre Research Ethics Committee (11/NW/0382) gave their ethical approval for the original UK Biobank study. Informed consent was obtained from each participant. Our access to data from the UK Biobank cohort was approved by the UKB Ethics Advisory Committee.

In late 2009, measurements of sensory function were started as an enhancement to baseline examinations. Visual acuity (VA) test was performed at six assessment centres (Croydon, Hounslow, Liverpool, Sheffield, Birmingham and Swansea) involving 117,175 participants, while Digit Triplet Test scores for determination of hearing ability were performed for 164,770 participants.

All participants were flagged from the date of their recruitment into the study with the National Health Service (NHS) Digital or NHS Central Register, and new data for death are currently provided every month and previously every three or six months to UK Biobank by these registries.

Inclusion criteria of the present study include having available data for speech-reception-threshold (SRT) and VA for both sides, 113,563 participants in total were included for further analysis.

### Definition of sensory impairments

For each participant, distant VA of both the left (UKB Field 5208–0.0) and right (UKB Field 5201–0.0) eyes were measured as the logarithm of the minimum angle of resolution (LogMAR), and VI was defined by the performance of a “better-seeing eye”. Participants were required to occlude the left eye and read letters from left to right and big to small on the LogMAR chart at a distance of 4 m. The individually test would be terminated when the participant identified 2 letters incorrectly. Then, the number of correctly identified letters was recorded as LogMAR VA. VI was defined as VA worse than LogMAR 0.3 [24, 25].

HI was defined by the performance of a “better ear” namely the more negative SRT score out of the left (UKB Field 20,019–0.0) and right (UKB Field 20,021–0.0) ear. The SRT was estimated through the Digit Triplet Test, which, in brief, is an automated hearing test on how well the participant can hear the signal (three spoken numbers, i.e., a digit triplet) played with a rushing noise in the background [26]. The signal-to-noise ratio (SNR) was defined as when half of the presented speech can be understood correctly. The noise level was altered to measure the signal to noise ratio (SNR) which was defined as the 50% correct recognition of the triplets, and SRT was calculated as the mean SNR of the last eight triplets. The standard for a ‘Normal’ score was -5.5 dB [27]. Accordingly, HI was defined as an SRT of over -5.5 dB in the participant’s better ear. Afterward, DSI was defined when one presented concurrent VI and HI. As the definitions of sensory impairments were well-established and widely used [28–30], misclassification of exposure was trivial.

### Ascertainment of death

Date of death (UKB Field 40,000–0.0) was obtained from NHS Digital for participants in England and Wales and the NHS Central Register, Scotland, for participants in Scotland. Participant follow-up started at inclusion in the UK Biobank study and ended either at the date of death, lost to follow-up or April 28, 2021.

### Covariates

Previously established risk factors were taken into consideration as covariates, including age (grouped into 40 ~ 50, 50 ~ 60, 60 ~), sex, ethnicity (White or others), education (a college/university degree or below), Townsend Index (continuous, positive values demonstrating more materially deprived), obesity (derived from whether a body mass index [BMI] ≥ 30 kg/m^2^), smoking status (never smoke, ex- or current smokers), physical activity (above moderate/vigorous/walking recommendation, or below), medical history (includes hyperlipidemia, hypertension, diabetes mellitus, cardiovascular diseases, cancer), and self-rated overall health status (excellent/good, fair/poor).

Medical history was majorly confined to self-reported or doctor-diagnosed diseases themselves. Hyperlipidemia was defined by having self-reported current statins or other hyperlipidemia-related medication, or cholesterol over 6.21 mmol/L. Hypertension was defined by having self-reported hypertension, current blood pressure treatment, systolic blood pressure over 130 mmHg, or diastolic blood pressure over 80 mmHg. Diabetes mellitus was defined by having prior doctor-diagnosed diabetes, current insulin therapy or other diabetes-related medication, or HbA1c >  = 48 mmol/mol (6.5%). Participants were asked “In general how would you rate your overall health?” for estimation of an overall health status (UKB Field 2178–0.0), and answers were recategorized into excellent/good and fair/poor.

### Statistical analysis

Participant characteristics were summarised using means and standard deviation for continuous variables and numbers and percentages for categorical variables. Comparisons of continuous variables between two groups (e.g., participants with and without VA&SRT data) and among more groups (e.g., NSI, VI-only, HI-only, and DSI) were done through unpaired t-test and ANOVA, respectively. Comparisons of categorical variables between groups were performed through Chi-Square Goodness-of-Fit Test. Associations between sensory impairments and mortality were analysed using two Cox proportional hazards model, and follow-up time was used as the underlying time scale. Model 1 adjusted for age and sex, while model 2 further adjusted for ethnicity, education, Townsend index, obesity, smoking status, physical activity, history of hyperlipidaemia, hypertension, diabetes and cancer, and overall health status. Relative risks were reported as hazard ratio (HR) with a 95% confidence interval (CI). All variables were assessed by Schoenfeld residuals to confirm validity (Additional file 2: Supplemental Table 2) and continuous variable was assessed by Martingale residual plots to check nonlinearity (Additional file 3: Supplemental Figure 1). As age group did not meet the proportional hazards assumption and non-linearity, subgroup analysis was done by age (40–50, 50–60, > 60). Subgroup analysis on gender was also performed. Three sensitivity analyses were conducted, excluding participants with a history of cancer, death within 1 year of inclusion, or death within 2 years of baseline assessment. Two-sided *P* value less than 0.05 accounted for statistical significance. Stata version 13 (StataCorp LLC, College Station, Texas USA) was used for all data analyses.

### Role of the funding source

The funders of the study had no role in study design, data collection, data analysis, data interpretation, or writing of the report. Corresponding authors had full access to all the data in the study and had final responsibility for the decision to submit for publication.

## Results

### Study population

After excluding 388,912 participants without measurements for VA or SRT, 113,563 participants with both VA and SRT records were included for analysis. Baseline characteristics of participants with or without VA/SRT record were compared in Additional file 2: Supplemental Table 3, and participants with VA/SRT records were older, less likely to be white, less materially deprived, have higher education level, less likely to be prior/current smokers, do more physical activities, more likely to have history of hyperlipidemia, hypertension, diabetes, cancer and have worse overall health status. Amongst the participants included, the mean age ± standard deviation (SD) was 56.8 ± 8.09 years, and 61,849 (54.5%) were female.

### Sensory impairment

Overall, 733 participants were identified with DSI, while 2,973 participants were found with single VI, 13,560 with single HI, and 96,297 reported neither sensory impairment (NSI) (Table 1). Compared with those of NSI, participants with any sensory impairment were more likely to be older, less likely to be White, have a higher Townsend index, have less than a college/university education, more likely to be obesity, more likely to do fewer physical activities, more likely to have any systematic disease history (includes hyperlipidemia, hypertension, diabetes, cancer, respectively), and have a fair/poor overall health status (Table 1). Additionally, participants with DSI were more likely to have the abovementioned characteristics than those with VI-/HI-only.

**Table 1:** Baseline characteristics stratified by sensory impairment status

Baseline characteristics	Total	NSI	VI-only	HI-only	DSI	*P* value
N	113,563	96,297	2,973	13,560	733	
Age, mean(SD), years	56.8(8.09)	56.3(8.08)	58.3(7.60)	59.9(7.51)	60.9(6.88)	** < 0.001**
Gender, N(%)						**0.026**
Female	61,849(54.5)	52,574(54.6)	1,649(55.5)	7,228(53.3)	398(54.3)	
Male	51,714(45.5)	43,723(45.4)	1,324(44.5)	6,332(46.7)	335(45.7)	
Ethnicity, N(%)						** < 0.001**
White	102,139(89.9)	88,283(91.7)	2,619(88.1)	10,690(78.8)	547(74.6)	
Others	11,424(10.1)	8,014(8.32)	354(11.9)	2,870(21.2)	186(25.4)	
Townsend Index, mean(SD)	-0.99(2.98)	-1.11(2.92)	-0.47(3.21)	-0.30(3.22)	0.48(3.43)	** < 0.001**
Education, N(%)						** < 0.001**
College/University degree	39,866(35.1)	35,123(36.5)	869(29.2)	3,728(27.5)	146(19.9)	
Without College/University degree	73,697(64.9)	61,174(63.5)	2,104(70.8)	9,832(72.5)	587(80.1)	
Obesity, N(%)						** < 0.001**
No	85,287(75.5)	72,902(76.1)	2,205(74.9)	9,682(72.0)	498(69.5)	
Yes	27,683(24.5)	22,961(24.0)	739(25.1)	3,764(28.0)	219(30.5)	
Smoking status, N(%)						0.146
Never	62,671(55.4)	53,271(55.5)	1,647(55.6)	7,373(54.7)	380(52.4)	
Prior/current	50,488(44.6)	42,733(44.5)	1,313(44.4)	6,097(45.3)	345(47.6)	
Above moderate/vigorous/walking recommendation, N(%)						**0.032**
No	16,386(17.6)	13,990(17.6)	404(16.9)	1,869(17.8)	123(22.0)	
Yes	76,516(82.4)	65,472(82.4)	1,994(83.2)	8,615(82.2)	435(78.0)	
History of hyperlipidemia, N(%)						** < 0.001**
No	61,024(53.7)	52,514(54.5)	1,587(53.4)	6,600(48.7)	323(44.1)	
Yes	52,539(46.3)	43,783(45.5)	1,386(46.6)	6,960(51.3)	410(55.9)	
History of hypertension, N(%)						** < 0.001**
No	29,195(25.7)	25,726(26.7)	656(22.1)	2,696(19.9)	117(16.0)	
Yes	84,368(74.3)	70,571(73.3)	2,317(77.9)	10,864(80.1)	616(84.0)	
History of diabetes mellitus, N(%)						** < 0.001**
No	106,223(93.5)	90,730(94.2)	2,751(92.5)	12,108(89.3)	634(86.5)	
Yes	7,340(6.46)	5,567(5.78)	222(7.47)	1,452(10.7)	99(13.5)	
History of cancer, N(%)						** < 0.001**
No	104,123(92.0)	88,524(92.2)	2,708(91.4)	12,232(90.6)	659(90.7)	
Yes	9,110(8.05)	7,513(7.82)	256(8.64)	1,273(9.43)	68(9.3)	
Overall health status, N(%)						** < 0.001**
Excellent/good	81,853(72.4)	70,720(73.7)	2,020(68.4)	8,684(64.6)	429(59.1)	
Fair/poor	31,274(27.6)	25,272(26.3)	935(31.6)	4,770(35.4)	297(40.9)	

*Abbreviations*: *NSI* Neither sensory impairment, *VI* Visual impairment, *HI* Hearing impairment, *DSI* Dual-sensory impairment, *SD* Standard deviation

### Mortality

Over a follow-up of 14 years (mean of 11 years), a total of 5,992 participants died from any causes. As shown in Additional file 2: Supplemental Table 4, deceased participants were significantly older (HR: 1.10 [95%CI, 1.09–1.11]), more likely to be male (HR: 1.66 [95%CI, 1.58–1.75]), with a lower Townsend index (HR: 1.07 [95%CI, 1.07–1.08]), were less likely to have a college/university education (HR: 1.33 [95%CI, 1.25–1.41]), were more likely to be obese (HR: 1.40 [95%CI, 1.33–1.48]), smoke (HR: 1.61 [95%CI, 1.53–1.70]), undertake less physical activity (HR: 0.67 [95%CI, 0.62–0.71], refer to above moderate/vigorous/walking recommendation) [31], have hyperlipidaemia (HR: 1.10 [95%CI, 1.05–1.10]), hypertension (HR: 1.21 [95%CI, 1.13–1.30]), diabetes (HR: 2.05 [95%CI, 1.91–2.21]), cancer (HR: 2.34 [95%CI, 2.20–2.50]), and worse overall health status (HR: 2.25 [95%CI, 2.13–2.36]).

### Association between sensory impairment and mortality

The Cox proportional hazards regression model suggested significant associations between mortality and DSI, VI-only or HI-only, after adjusting for age and sex (Table 2). Number of individuals who died after follow-up for specific reasons and person-years in each group were demonstrated in Additional file 2: Supplemental Table 5. Further, the multivariable regression model, adjusted for confounding factors (ethnicity, education, Townsend index, obesity, smoking status, physical activity, history of hyperlipidaemia, hypertension, diabetes and cancer, and overall health status), indicated that participants with DSI were at an estimated 44% higher risk of mortality compared to NSI (HR: 1.44 [95%CI, 1.11–1.88], *p* = 0.007). Single VI or HI was also significantly associated with a higher mortality risk compared to NSI (VI-only: HR, 1.26 [95%CI, 1.07–1.48], *p* = 0.005; HI-only: HR, 1.23 [95%CI, 1.14–1.33], *p* < 0.001) (Table 2).

**Table 2:** Association between sensory impairment status and all-cause mortality

Sensory Impairment status	Age- and gender-adjusted model		Multivariable model^a^		Number of deceased participants	Person-year of deceased participants
	HR (95% CI)	*P* value	HR (95% CI)	*P* value		
Main analysis						
NSI	1[Reference]		1[Reference]		4,579	3.86 × 10^8^
VI-only	1.33 (1.16–1.52)	** < 0.001**	1.26 (1.07–1.48)	**0.005**	213	1.18 × 10^7^
HI-only	1.33 (1.24–1.42)	** < 0.001**	1.23 (1.14–1.33)	** < 0.001**	1,124	5.34 × 10^7^
DSI	1.59 (1.27–2.00)	** < 0.001**	1.44 (1.11–1.88)	**0.007**	76	2.84 × 10^6^
Sensitivity analysis^b^						
NSI	1[Reference]		1[Reference]		3,707	3.56 × 10^8^
VI-only	1.34 (1.15–1.56)	** < 0.001**	1.20 (1.00–1.44)	0.051	169	1.08 × 10^7^
HI-only	1.42 (1.32–1.52)	** < 0.001**	1.25 (1.15–1.36)	** < 0.001**	900	4.87 × 10^7^
DSI	1.79 (1.39–2.29)	** < 0.001**	1.54 (1.15–2.06)	**0.004**	63	2.59 × 10^6^
Sensitivity analysis^c^						
NSI	1[Reference]		1[Reference]		4,434	3.85 × 10^8^
VI-only	1.37 (1.19–1.57)	** < 0.001**	1.26 (1.07–1.48)	**0.006**	206	1.18 × 10^7^
HI-only	1.42 (1.33–1.52)	** < 0.001**	1.24 (1.15–1.34)	** < 0.001**	1,094	5.34 × 10^7^
DSI	1.68 (1.33–2.12)	** < 0.001**	1.43 (1.09–1.88)	**0.009**	71	2.84 × 10^6^
Sensitivity analysis^d^						
NSI	1[Reference]		1[Reference]		4,190	3.84 × 10^8^
VI-only	1.38 (1.20–1.60)	** < 0.001**	1.26 (1.07–1.49)	**0.006**	197	1.18 × 10^7^
HI-only	1.42 (1.33–1.52)	** < 0.001**	1.25 (1.15–1.35)	** < 0.001**	1,034	5.34 × 10^7^
DSI	1.62 (1.27–2.07)	** < 0.001**	1.38 (1.04–1.83)	**0.026**	65	2.84 × 10^6^

*Abbreviations*: *NSI* Neither sensory impairment, *VI* Visual impairment, *HI* Hearing impairment, *DSI* Dual-sensory impairment, *HR* Hazard ratio, *CI* Confidence interval

^a^ Adjusted for age, gender, ethnicity, education, Townsend index, obesity, smoking status, physical activity, history of hyperlipidemia, hypertension, diabetes and cancer, and overall health status

^b^ Participants with history of cancer at the baseline assessment were excluded

^c^ Deaths recorded within one year after the baseline assessment were excluded

^d^ Deaths recorded within two years after the baseline assessment were excluded

In the first sensitivity analysis which excluded participants with a history of cancer at the baseline assessment, associations between DSI, single HI and mortality (compared to no impairment) were in the same direction with stronger magnitudes in the fully adjusted multivariable model compared to the original population (DSI: HR 1.54 [95%CI, 1.15–2.06], *p* = 0.004; HI-only: HR 1.25 [95%CI, 1.15–1.36], *p* < 0.001) (Table 2). For VI-only in this model, although the direction was the same, that no significant association was found. DSI, or any single sensory impairment were associated with higher risks of mortality after excluding participants who died within one year of baseline examination after multiple adjustments (DSI: HR 1.43 [95%CI, 1.09–1.88], *p* = 0.009) (Table 2). And the same results were found after excluding those who died within 2 years of the baseline examination (HR 1.38 [95%CI, 1.04–1.83], *p* < 0.001) (Table 2).

In the subgroup analysis of age after multiple adjustments, the same trend was found in participants aged over 60. Participants with DSI had 42% higher risk of mortality compared with NSI, while VI and HI were at 25% and 26% higher risk, respectively (DSI: HR 1.42 [95%CI, 1.06–1.90], *p* = 0.020; VI-only: HR 1.25 [95%CI, 1.04–1.51], *p* < 0.001; HI-only: HR 1.26 [95%CI, 1.16–1.38], *p* < 0.001). In the subgroup analysis of gender after multiple adjustment, participants with DSI were only found at higher risk in female subgroup (HR 1.65 [95%CI, 1.11–2.45], *p* = 0.014), those with VI were only found at higher risk in male subgroup (HR 1.25 [95%CI, 1.02–1.54], *p* = 0.035), and both female and male with HI were at higher risk of mortality (female: HR 1.19 [95%CI, 1.04–1.36], *p* = 0.009; male: HR 1.25 [95%CI, 1.14–1.38], *p* < 0.001). In the subgroup analysis of follow-up period, VI only (HR 1.28 [95%CI, 1.02–1.60], *p* = 0.032) and HI only (HR 1.33 [95%CI, 1.19–1.48], *p* < 0.001) were found significantly associated with mortality in those who have longer follow-up (over P50, which was 7.05 years), and none was found significantly associated with mortality in subgroup of shorter follow-up time. (Additional file 2: Supplemental Table 6).

## Discussion

In the present study, we investigated the association of DSI, VI and HI with the long-term risk of mortality using a large population-based sample from the UK Biobank study, over a maximum duration of follow-up of 14 years. After adjustment for confounding factors (includes demographic, socioeconomic characteristics, lifestyle factors and clinical factors), we identified that DSI would significantly increase the risk of mortality by 44% when compared with NSI. Participants with single VI and single HI had 1.3- and 1.2-fold increased risk of mortality, respectively.

This study explored the positive association between DSI and mortality in a large national sample of European adults across a wide range of ages. Notably, in the subgroup analysis, we found the trend remain significant in female and participants aged 60 and older. Our findings are supported by prior studies [15–20]. Most of the prior studies with insignificant associations included mainly participants over 50 years old, while only two prior studies were conducted with adults aged 18 and over recruited from the National Health Interview Survey (NHIS) and reported an increased risk of mortality in people with DSI [15, 20]. Lam and colleagues reported a significantly increased risk of mortality associated with DSI amongst Caucasians and other races, yet this association was not observed in African Americans [15]. Our study also included participants of different races and included adjustments for ethnicity as a potential confounder. However, we lacked the power to investigate the association of DSI and mortality within a single race, such as African Americans, due to small sample sizes in these subgroups. Lee et al. indicated a higher risk of mortality with higher severity of DSI over 8 years of follow-up, while the association were weaker for participants with only moderate/mild single VI or HI [20]. This is consistent with our findings that individuals with DSI had a higher risk of mortality compared with those with a single sensory impairment.

There are several possible underlying mechanisms for the observed association between DSI and mortality. For instance, impairment of sensory functions directly interferes with activities of daily living, leading to reduced mobility, increased rate of depression and social deprivation, barriers to healthcare services, and finally increased vulnerability to life-threatening accidents [32–36]. Individuals with DSI are even more restricted than those with a single sensory impairment, as they are not able to use the unimpaired sensory function as a compensation [37]. Alternatively, VI and HI may be viewed as indicators of ageing [38, 39]. Since a variety of causes for VI and HI (e.g., cataract, presbycusis) are strongly age-related, sensory dysfunction may signify an accelerated process of biological ageing [13, 14, 40]. In such circumstances, DSI may indicate a more advanced status of aging, and a more severe degenerative process. Finally, it is also suggestive that some mutual underlying mechanisms are responsible for both systematic diseases and diseases leading to sensory impairments, attributed as "common causes", such as oxidative stress, microvascular changes, and chronic inflammation, etc. [41–43].

The strength of this study includes a large sample of representative adult participants from the UK Biobank study, which increase the generalisability of our findings. In addition, the long follow-up period up to a maximum of 14 years allowed us to comprehensively explore the long-term effect of sensory impairments on mortality. This study provides insight on the public policy to attach importance to prevent avoidable sensory impairments through addressing common reasons for sensory impairments. It also expands on the work of prior studies by using standardised objective measurements for sensory functions and universal criteria for VI and HI. Nevertheless, our results are limited by the following elements: 1, there are quantitative confounding factors affecting mortality, while this study has included as many measurable covariates as possible for a comprehensive adjustment it is possible that there are residual confounding factors that we were unable to account for. 2, the relatively small number of DSI individuals who died during follow up (*n* = 76) which limited further sub-group analysis. 3, we were unable to analyse the association between mortality and different ocular and acoustic diseases as causes for sensory impairment due to limited sample size of deceased participants with DSI. 4, though the definition of visual and hearing impairments in the present study were widely adopted, there may still be some cases of misclassification within acceptable deviation. 5, our study is about the association between baseline sensory impairment status with mortality, so future study with regular follow-up of visual acuity and hearing status are called for to see how longitudinal sensory changes contributes to mortality.

## Conclusions

This study included a large nationally representative sample from the UK Biobank and provides evidence that individuals with DSI were at a 44% higher risk of mortality than NSI, independent of age, sex, and other confounders. Further research and public policies are warranted to prevent avoidable sensory impairment and rescue function of sensory systems for population with a high risk of mortality.

## Supplementary Information

**Additional file 1:** Supplemental Table 1.

**Additional file 2:** Supplemental Tables 2-6.

**Additional file 3:** Supplemental Figure 1.

## Data Availability

The dataset analysed during the present study is available in the UK Biobank (https://www.ukbiobank.ac.uk/). These data are not publicly available because they were analysed under licence, but they are available from the corresponding author on reasonable request and with permission of UK Biobank.

## Abbreviations

VI: Visual impairment
HI: Hearing impairment
DSI: Dual sensory impairment
VA: Visual acuity
NHS: National Health Service
SRT: Speech-reception-threshold
LogMAR: Logarithm of the minimum angle of resolution
SNR: Signal to noise ratio
BMI: Body mass index
HR: Hazard ratio
CI: Confidence interval
SD: Standard deviation
NHIS: National Health Interview Survey
