# Correction: Association between dual sensory impairment and risk of mortality: a cohort study from the UK Biobank

**DOI:** 10.1186/s12877-022-03384-x

**Published:** 2022-08-26

**Authors:** Xinyu Zhang, Yueye Wang, Wei Wang, Wenyi Hu, Xianwen Shang, Huan Liao, Yifan Chen, Katerina V. Kiburg, Yu Huang, Xueli Zhang, Shulin Tang, Honghua Yu, Xiaohong Yang, Mingguang He, Zhuoting Zhu

**Affiliations:** 1 Department of Ophthalmology, Guangdong Academy of Medical Sciences, Guangdong Provincial People’s Hospital, Guangzhou, China; 2 Department of Ophthalmology, Shanghai General Hospital, Shanghai, China; 3 State Key Laboratory of Ophthalmology, Zhongshan Ophthalmic Center, Sun Yat-Sen University, Guangzhou, China; 4 Centre for Eye Research, University of Melbourne, East Melbourne, Victoria Australia; 5 Neural Regeneration Group, Institute of Reconstructive Neurobiology, University of Bonn, Bonn, Germany; 6 John Radcliffe Hospital, Oxford University Hospitals NHS Foundation Trust, Oxford, UK

**Correction: BMC Geriatrics 22, 631 (2022)**

**https://doi.org/10.1186/s12877-022-03322-x**

After publication of this article [1], the authors reported that in the Funding information section, a funding granted to Mingguang He was left out by mistake. He was supported by the ‘High-level Talent Flexible Introduction Fund of Guangdong Provincial People’s Hospital (No. KJ012019530)’.

The original article [1] has been corrected.
