# Supplementary material for: Association between dual sensory impairment and risk of mortality: a cohort study from the UK Biobank
Source: BMC Geriatr. 2022 Aug 1;22:631. doi: 10.1186/s12877-022-03322-x (PMC9341066; doi:10.1186/s12877-022-03322-x)

**Supplementary material**

| Supplemental Table 2. P values of Schoenfeld residuals for all variables. | | | | |  |
| --- | --- | --- | --- | --- | --- |
| Variables | Main Analysis | Subgroup analysis on Age | | | |
|  |  | 40-50 | 50-60 | >60 | |
| Age (50-60) | **0.033** | - | - | - | |
| Age (>60) | **<0.001** | - | - | - | |
| Gender (Males) | 0.084 | 0.540 | 0.083 | 0.272 | |
| Ethnicity (Others) | 0.175 | 0.284 | 0.477 | 0.616 | |
| Townsend Index | 0.175 | 0.322 | 0.363 | 0.197 | |
| Education (Without College/University degree) | 0.502 | 0.873 | 0.892 | 0.424 | |
| Obesity (Yes) | 0.594 | 0.359 | 0.778 | 0.625 | |
| Smoking status (Prior/current) | 0.085 | 0.652 | 0.202 | **0.004** | |
| Above moderate/vigorous/walking recommendation (Yes) | 0.098 | 0.888 | 0.136 | 0.210 | |
| History of hyperlipidemia (Yes) | 0.450 | 0.504 | 0.572 | 0.671 | |
| History of hypertension (Yes) | 0.225 | 0.540 | 0.025 | 0.961 | |
| History of diabetes mellitus (Yes) | 0.283 | 0.393 | 0.498 | 0.207 | |
| Overall health status (Fair/poor) | 0.049 | 0.362 | 0.129 | 0.222 | |

| Supplemental Table 3. Baseline characteristics stratified by the availability of VA and (or) SRT data | | | |
| --- | --- | --- | --- |
| Baseline characteristics | Participants without VA and (or) SRT data | Participants with VA and SRT data | P value |
| N | 388,912 | 113,563 |  |
| Age, mean(SD), years | 56.4 (8.09) | 56.8 (8.09) | **<0.001** |
| Gender, N(%) |  |  | 0.652 |
| Female | 211,516(54.4) | 61,849(54.5) |  |
| Male | 177,396(45.6) | 51,714(45.5) |  |
| Ethnicity, N(%) |  |  | **<0.001** |
| White | 370,529(95.3) | 102,139(89.9) |  |
| Others | 18,384(4.7) | 11,424(10.1) |  |
| Townsend Index, mean(SD) | -1.38(3.12) | -0.99(2.98) | **<0.001** |
| Education, N(%) |  |  | **<0.001** |
| College/University degree | 121,286(31.2) | 39,866(35.1) |  |
| Without College/University degree | 267,627(68.8) | 73,697(64.9) |  |
| Obesity, N(%) |  |  | 0.818 |
| No | 291,844(75.5) | 85,287(75.5) |  |
| Yes | 94,557(24.5) | 27,683(24.5) |  |
| Smoking status, N(%) |  |  | **<0.001** |
| Never | 210,833(54.6) | 62,671(55.4) |  |
| Prior/current | 175,535(45.4) | 50,488(44.6) |  |
| Above moderate/vigorous/walking recommendation, N(%) |  |  | **<0.001** |
| No | 58,198(18.8) | 16,386(17.6) |  |
| Yes | 251,148(81.2) | 76,516(82.4) |  |
| History of hyperlipidemia, N(%) |  |  | **<0.001** |
| No | 211,627(54.4) | 61,024(53.7) |  |
| Yes | 177,286(45.6) | 52,539(46.3) |  |
| History of hypertension, N(%) |  |  | **<0.001** |
| No | 109,982(28.3) | 29,195(25.7) |  |
| Yes | 278,931(71.7) | 84,368(74.3) |  |
| History of diabetes mellitus, N(%) |  |  | **<0.001** |
| No | 365,993(94.1) | 106,223(93.5) |  |
| Yes | 22,920(5.89) | 7,340(6.46) |  |
| History of cancer, N(%) |  |  | **<0.001** |
| No | 356,963(92.4) | 104,123(92.0) |  |
| Yes | 29,504(7.63) | 9,110(8.05) |  |
| Overall health status, N(%) |  |  | **<0.001** |
| Excellent/good | 289,003(74.9) | 81,853(72.4) |  |
| Fair/poor | 96,859(25.1) | 31,274(27.6) |  |
| Abbreviations: VA=visual acuity, SRT=speech-reception threshold, SD=standard deviation | | | |

| Supplemental Table 4. Baseline characteristics stratified by all-cause mortality. | | | | | |
| --- | --- | --- | --- | --- | --- |
| Baseline characteristics | Living participants | Deceased participants | P value | HR (95% CI)^a^ | P value |
| N | 107,571 | 5,992 |  |  |  |
| Age, mean(SD), years | 56.5(8.09) | 61.8(6.26) | **<0.001** | 1.10(1.09-1.11) | **<0.001** |
| Gender, N(%) |  |  | **<0.001** |  |  |
| Female | 59,393(55.2) | 2,456(41.0) |  | 1[Reference] |  |
| Male | 48,178(44.8) | 3,536(59.0) |  | 1.66(1.58-1.75) | **<0.001** |
| Ethnicity, N(%) |  |  | **<0.001** |  |  |
| White | 96,568(89.8) | 5,571(93.0) |  | 1[Reference] |  |
| Others | 11,003(10.2) | 421(7.03) |  | 0.94(0.85-1.04) | 0.238 |
| Townsend Index, mean(SD) | -1.01(2.97) | -0.57(3.16) | **<0.001** | 1.07(1.07-1.08) | **<0.001** |
| Education, N(%) |  |  | **<0.001** |  |  |
| College/University degree | 38,289(35.6) | 1,577(26.3) |  | 1[Reference] |  |
| Without College/University degree | 69,282(64.4) | 4,415(73.7) |  | 1.33(1.25-1.41) | **<0.001** |
| Obesity, N(%) |  |  | **<0.001** |  |  |
| No | 81,232(75.9) | 4,055(68.5) |  | 1[Reference] |  |
| Yes | 25,815(24.1) | 1,868(31.5) |  | 1.40(1.33-1.48) | **<0.001** |
| Smoking status, N(%) |  |  | **<0.001** |  |  |
| Never | 60,293(56.2) | 2,378(39.9) |  | 1[Reference] |  |
| Prior/current | 46,906(43.8) | 3,582(60.1) |  | 1.61(1.53-1.70) | **<0.001** |
| Above moderate/vigorous/walking recommendation, N(%) |  |  | **<0.001** |  |  |
| No | 15,316(17.4) | 1,070(22.9) |  | 1[Reference] |  |
| Yes | 72,904(82.6) | 3,612(77.1) |  | 0.67(0.62-0.71) | **<0.001** |
| History of hyperlipidemia, N(%) |  |  | **<0.001** |  |  |
| No | 58,389(54.3) | 2,635(44.0) |  | 1[Reference] |  |
| Yes | 49,182(45.7) | 3,357(56.0) |  | 1.10(1.05-1.10) | **<0.001** |
| History of hypertension, N(%) |  |  | **<0.001** |  |  |
| No | 28,249(26.3) | 946(15.8) |  | 1[Reference] |  |
| Yes | 79,322(73.7) | 5,046(84.2) |  | 1.21(1.13-1.30) | **<0.001** |
| History of diabetes mellitus, N(%) |  |  | **<0.001** |  |  |
| No | 101,132(94.0) | 5,091(85.0) |  | 1[Reference] |  |
| Yes | 6,439(5.99) | 901(15.0) |  | 2.05(1.91-2.21) | **<0.001** |
| History of cancer, N(%) |  |  | **<0.001** |  |  |
| No | 99,302(92.6) | 4,821(80.7) |  | 1[Reference] |  |
| Yes | 7,957(7.42) | 1,153(19.3) |  | 2.34(2.20-2.50) | **<0.001** |
| Overall health status, N(%) |  |  | **<0.001** |  |  |
| Excellent/good | 78,610(73.4) | 3,243(54.5) |  | 1[Reference] |  |
| Fair/poor | 28,561(26.6) | 2,713(45.5) |  | 2.25(2.13-2.36) | **<0.001** |
| Abbreviations: SD=standard deviation, HR=hazard ratio, CI=confidence interval | | | |  |  |
| ^a^ Age- and gender-adjusted | | | |  |  |

Supplemental Table 5. Number of deaths and person-years after follow-up.

| Sensory impairment status | Number of deaths | | | | Person-years |
| --- | --- | --- | --- | --- | --- |
|  | Cancer-specific death | CVD-specific death | Non-cancer and non-CVD death | Total |  |
| NSI | 2,511 | 811 | 1,257 | 4,579 | 3.86×10^8^ |
| VI-only | 95 | 37 | 81 | 213 | 1.18×10^7^ |
| HI-only | 524 | 225 | 375 | 1124 | 5.34×10^7^ |
| DSI | 29 | 17 | 30 | 76 | 2.84×10^6^ |
| Abbreviations: CVD=cardiovascular disease, NSI=neither sensory impairment, VI=visual impairment, HI=hearing impairment, DSI=dual-sensory impairment, SD=standard deviation | | | | | |

| Supplemental Table 6. Subgroup analysis on association between sensory impairment status and all-cause mortality. | | | | | | | | |
| --- | --- | --- | --- | --- | --- | --- | --- | --- |
| Sub-group Analysis^a^ | NSI | | VI | | HI | | DSI | |
|  | HR (95% CI) | P value | HR (95% CI) | P value | HR (95% CI) | P value | HR (95% CI) | P value |
| Age |  |  |  |  |  |  |  |  |
| 40-50 | 1[Ref] | - | 2.73(1.56, 4.78) | **<0.001** | 1.13(0.702, 1.83) | 0.606 | 3.05(0.755, 12.3) | 0.118 |
| 50-60 | 1[Ref] | - | 0.991(0.680, 1.45) | 0.963 | 1.12(0.904, 1.39) | 0.299 | 1.46(0.723, 2.93) | 0.293 |
| >60 | 1[Ref] | - | 1.25(1.04, 1.51) | **0.017** | 1.26(1.16, 1.38) | **<0.001** | 1.42(1.06, 1.90) | **0.020** |
| Gender |  |  |  |  |  |  |  |  |
| Female | 1[Ref] | - | 1.26(0.980, 1.61) | 0.072 | 1.19(1.04, 1.36) | **0.009** | 1.65(1.11, 2.45) | **0.014** |
| Male | 1[Ref] | - | 1.25(1.02, 1.54) | **0.035** | 1.25(1.14, 1.38) | **<0.001** | 1.30(0.913, 1.86) | 0.144 |
| Abbreviations: NSI=neither sensory impairment, VI=visual impairment, HI=hearing impairment, DSI=dual-sensory impairment, HR=hazard ratio, CI=confidence interval | | | | | | | | |
| ^a^ Adjusted for age, gender, ethnicity, education, Townsend index, obesity, smoking status, physical activity, history of hyperlipidemia, hypertension, diabetes and cancer, and overall health status. | | | | | | | | |

Supplemental Figure 1. Martingale residual for continuous variable.


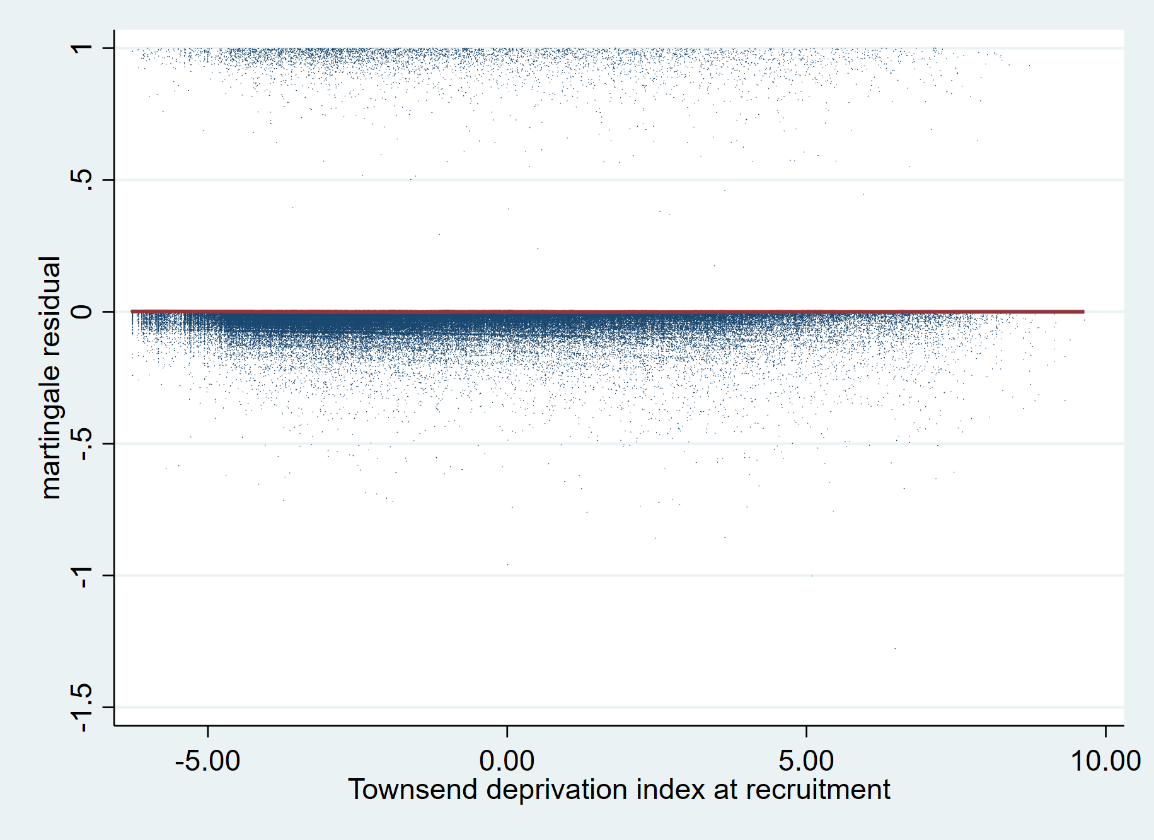

Supplement: Supplementary file 2 — Additional file 2: Supplemental Tables 2-6. [file 12877_2022_3322_MOESM2_ESM.docx]
