# Supplementary figures and images for: Association between dual sensory impairment and risk of mortality: a cohort study from the UK Biobank
Source: BMC Geriatr. 2022 Aug 1;22:631. doi: 10.1186/s12877-022-03322-x (PMC9341066; doi:10.1186/s12877-022-03322-x)

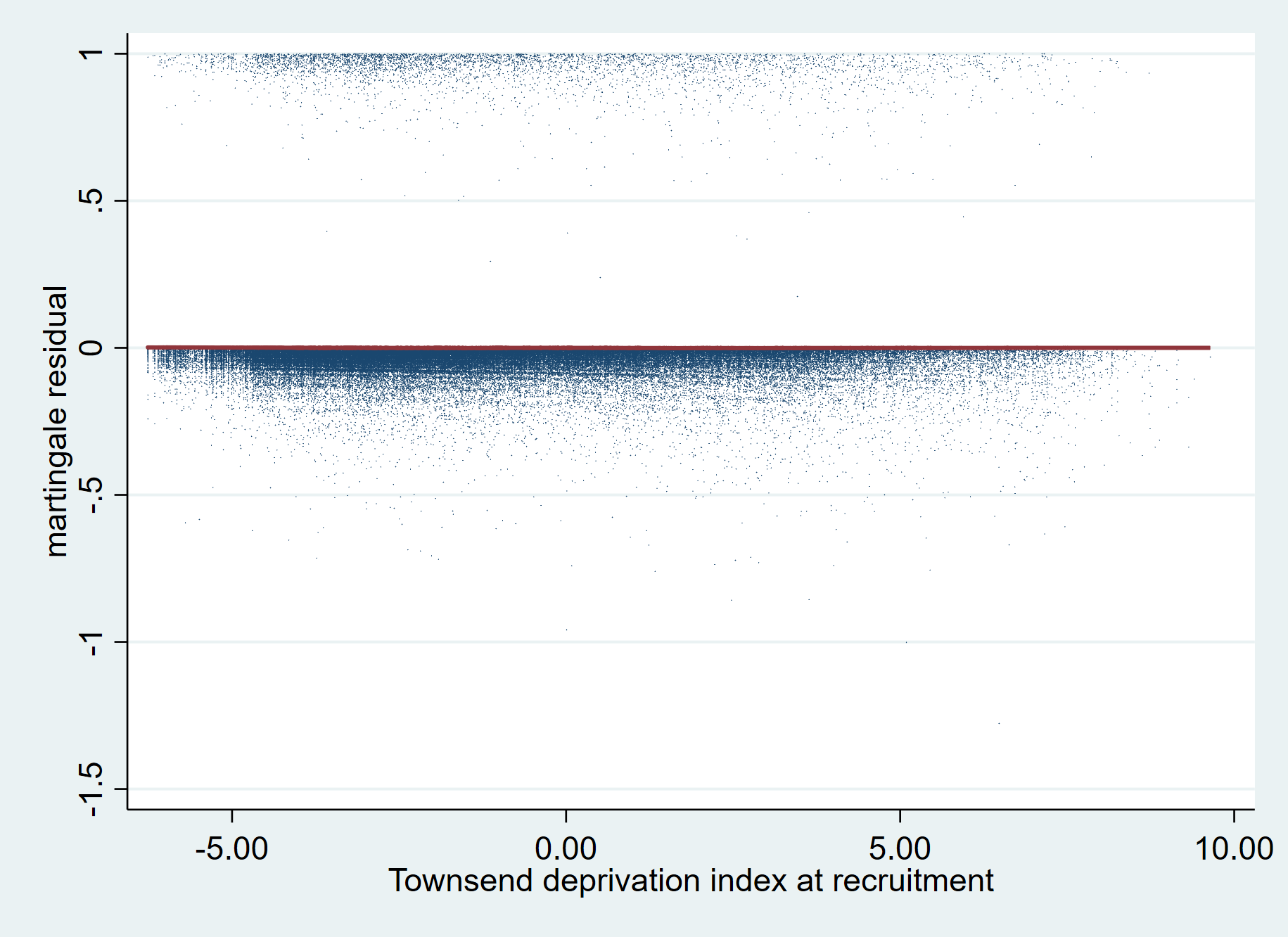

Supplement: Supplementary file 3 — Additional file 3: Supplemental Figure 1. [file 12877_2022_3322_MOESM3_ESM.tif]
